# A Lattice-Boltzmann scheme for the simulation of diffusion in intracellular crowded systems

**DOI:** 10.1186/s12859-015-0769-8

**Published:** 2015-11-03

**Authors:** Liliana Angeles-Martinez, Constantinos Theodoropoulos

**Affiliations:** School of Chemical Engineering and Analytical Science, University of Manchester, Manchester, M13 9PL UK

**Keywords:** Lattice Boltzmann Method, Scaled Particle Theory, Crowding conditions, Diffusion systems

## Abstract

**Background:**

The intracellular environment is a complex and crowded medium where the diffusion of proteins, metabolites and other molecules can be decreased. One of the most popular methodologies for the simulation of diffusion in crowding systems is the Monte Carlo algorithm (MC) which tracks the movement of each particle. This can, however, be computationally expensive for a system comprising a large number of molecules. On the other hand, the Lattice Boltzmann Method (LBM) tracks the movement of collections of molecules, which represents significant savings in computational time. Nevertheless in the classical manifestation of such scheme the crowding conditions are neglected.

**Methods:**

In this paper we use Scaled Particle Theory (SPT) to approximate the probability to find free space for the displacement of hard-disk molecules and in this way to incorporate the crowding effect to the LBM. This new methodology which couples SPT and LBM is validated using a kinetic Monte Carlo (kMC) algorithm, which is used here as our "computational experiment".

**Results:**

The results indicate that LBM over-predicts the diffusion in 2D crowded systems, while the proposed coupled SPT-LBM predicts the same behaviour as the kinetic Monte Carlo (kMC) algorithm but with a significantly reduced computational effort. Despite the fact that small deviations between the two methods were observed, in part due to the mesoscopic and microscopic nature of each method, respectively, the agreement was satisfactory both from a qualitative and a quantitative point of view.

**Conclusions:**

A crowding-adaptation to LBM has been developed using SPT, allowing fast simulations of diffusion-systems of different size hard-disk molecules in two-dimensional space. This methodology takes into account crowding conditions; not only the space fraction occupied by the crowder molecules but also the influence of the size of the crowder which can affect the displacement of molecules across the lattice system.

**Electronic supplementary material:**

The online version of this article (doi:10.1186/s12859-015-0769-8) contains supplementary material, which is available to authorized users.

## Background

Several microorganisms are used in the conversion of raw materials to added-value products, for example *Actinobacillus succinogenes* has been used for the synthesis of succinic acid from crude bio refinery glycerol [1, 2]. The analysis and simulation of the factors affecting the metabolism of these organisms allow the further identification of the strategies needed for its manipulation in order to increase the formation of the metabolite of interest over other by-products.

As it is known the environmental conditions and the properties of the medium play an important role in the metabolism. The intracellular processes are carried out in a complex, heterogeneous, and crowded medium composed by solid components (macromolecules, enzymes, etc.) in a fluid phase called cytoplasm (in 3D) or in cell membranes (2D) [3, 4], where for prokaryotes the diffusion is the primary mean of intracellular motion.

According to a drawing proposed by Goodsell [5], if the cytoplasm of *Escherichia coli* is divided into 600 cubes of (100 nm)^3^, an average of 130 glycolytic enzymes and 100 from the Krebbs cycle are present in each cube in addition to the metabolites and other compounds, which all together comprise a very large number of molecules for the simulation of the intracellular environment. Henceforth, we will use the terms *molecule* and *particle* interchangeably to refer to the intracellular macromolecules, e.g., proteins.

The solid components of the cell occupy about 40 % of the total volume [6] and 25 % of the area of a typical membrane [7]. Due to the impossibility that two molecules occupy the same space at the same time (steric effects), these *crowding* conditions affect the intracellular process in two opposite ways: 1) decreasing the diffusion of the molecules [8], and 2) increasing the thermodynamic activity of the metabolites [6], and therefore enhancing the reaction rate, and modifying the thermodynamic feasibility of some reactions. A review of the crowding effects can be found in [9].

In particular the study and simulation of the diffusion process can be carried out using several methodologies at different levels of detail. One of the most popular is the Monte Carlo (MC) algorithm [10–17] (microscopic level) where each molecule is tracked during its journey through the cell so the restriction of the impenetrability of the molecules is satisfied in a straightforward way.

MC is a powerful technique and easy to implement, however it is limited to short simulation times, restricted lattice/domain sizes, and/or reduced number of molecules because of the large computational costs. Besides, due to the stochastic nature of MC, it requires several simulations to smoothen the noise of the results by computing average quantities. Moreover, in most cases the molecules are considered to be of the same size, so the size effect could be hidden [10, 11].

On the other hand, Lattice Boltzmann (LBM) [18] is a mesoscopic method which allows the efficient simulation of the dynamics of collections across a defined lattice according to expressions that conserve mass and momentum [19]. Here, the solute transport is simulated either 1) treating the solute as another fluid and solving a multicomponent problem (active solute component) or 2) assuming that the molecules do not have velocity of their own so they are advected by the fluid (passive solute component). In both cases the volume of the solute is neglected. See Sukop and Thorne [20] for a review.

Alternatively, in particle suspensions simulations the motion of each molecule is described by a hard sphere model (with the drawback of being computationally expensive for large numbers of particles) while the fluid is described by LBM [21, 22]. This is similar to other hybrid methods used for example to follow the enzymes’ motion with MC, or the tumour growth with Cellular Automata [23], while the passive transport of the metabolites and the fluid is simulated by LBM.

Since LBM computes the evolution of the average molecules’ density, it represents a good alternative to simulate the diffusion of large number of intracellular macromolecules or even metabolites. However, since classical LBM does not take into account the volume of the molecules, and therefore the effect of obstacles on the molecules’ diffusion, it may overestimate the degree of mixing of the system analysed.

The displacement of a molecule depends on the probability *P* to find enough empty space to move at every step of its journey. Scaled Particle Theory (SPT) is a method that allows the estimation of this probability *P* which is a function of the radii and concentration/number of molecules present in the system. SPT also has been used to investigate the effect of macromolecular crowding on solvation [24], thermodynamic activity of proteins [25] and of metabolites [26].

The aim of this paper is to incorporate the crowding effect on the LBM simulation of the particles’ diffusion. For this, a methodology is proposed for coupling SPT and LBM, allowing in this way faster simulations for systems with a large number of molecules of different size under crowded conditions, such as the intracellular environment. Here, we consider the diffusing molecules as passive solute components assuming that the fluid phase is at rest within the cell. In particular, this paper focuses on 2D simulations of macromolecules’ diffusion which are relevant for the study and analysis of lateral diffusion of proteins in membranes [27–29]. For validation purposes the results are compared with those obtained from kinetic Monte Carlo (kMC) algorithm [14, 15].

## Methods

In the classical LBM [18], the system is represented by a regular lattice, where the molecules located at the same site or node at time *t* may interact with each other (collision step), and then according to a set of rules, some particles move to one of their neighbouring lattice sites (known as the streaming step), where they will interact with molecules from other nodes at time *t* + Δ*t* and so on.

The methodology we propose here corrects the average number of molecules that enter a neighbouring lattice site, taking into account crowding effects and it can be summarised as follows:

1. Solve the classical LBM to find the number of molecules that will try to move into the *d* direction at time *t* + Δ*t* (*F*_*d*_^*LB*^).
2. Use *F*_*d*_^*LB*^ to estimate the corrected number of molecules that actually enter the target site, *F*_*d*_, by solving the explicit formulation (see below) constrained by the size and number of the molecules, as well as the size of voxels or sites in which the lattice is divided.
3. Use the *F*_*d*_ values obtained in (2) for the streaming step (in the same way as in the classical LBM), and go back to point (1).

Here we use the term crowding-Lattice Boltzmann Method (cLBM) to distinguish this proposed methodology from the classical LBM, which in principle considers volumeless molecules.

### Crowding-Lattice Boltzmann Method (cLBM)

#### 1. Lattice Boltzmann Method

**Fig. 1:**
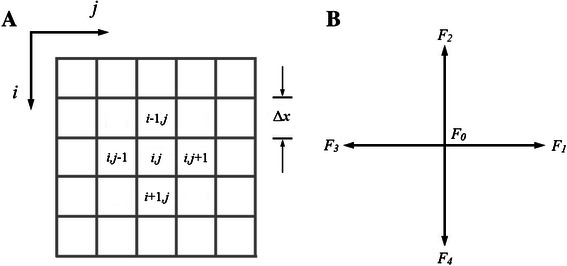
**a** Lattice scheme and (**b**) D2Q5 scheme

For comparison purposes with the kMC algorithm, in this paper the D2Q5 scheme [23] (Fig. 1b) is implemented, consisting of 5 possible directions in which the molecules can move, in a 2D lattice. The lattice is divided in squares of Δ*x* [nm] side, called “voxels”, whose position is identified by the index (*i,j*) (Fig. 1a).

The evolution of the distribution function of the species *sp, F*_*d,sp*_, in the diffusion system is given by the discrete Boltzmann equation [19].

1 $$ {F}_{d,sp}^{LB}\kern0.28em \left({i}_{next},{j}_{next},t+\Delta t\right)={F}_{d,sp}\kern0.28em \left(i,j,t\right)+{\Omega}_{d,sp}^{diff}\kern0.28em \left(i,j,t\right) $$

The superscript LB is used to distinguish *F*_*d*,*sp*_^*LB*^ that is calculated from the classical LBM from the crowding-corrected value *F*_*d,sp*_. Both *F*_*d*,*sp*_^*LB*^ and *F*_*d,sp*_ represent the concentration or number of molecules of the species *sp* in a voxel that try to move to a neighbouring site, so they are given in [molecules per voxel]. Other concentration units e.g., [mol per voxel] can be used, but dimensional changes in other variables are required for consistency.

We use the BGK approximation to estimate the non-reactive collision term Ω_*d*,*sp*_^*diff*^, which is given by [19].

2 $$ {\Omega}_{d,sp}^{diff}\left(i,j,t\right)={\omega}_{sp}\left[{F}_{d,sp}^{eq}\left(i,j,t\right)-{F}_{d,sp}\left(i,j,t\right)\right] $$

Assuming that the fluid phase is at rest, the equilibrium distribution function *F*_*d*,*sp*_^*eq*^ takes the form [23]

3 $$ {F}_{d,sp}^{eq}\;\left(i,j,t\right)=\frac{\rho_{sp}\;\left(i,j,t\right)}{m_{sp}}{w}_d $$

where the weight factor *w*_*d*_ is 0 for *d* = 0, and 1/4 for *d* = 1,2,3,4, while *m*_*sp*_ is the mass of one single molecule of type *sp*. The macroscopic density of species *sp*, *ρ*_*sp*_, is expressed as:

4 $$ {\rho}_{sp}\;\left(i,j,t\right)={m}_{sp}{\displaystyle \sum_d{F}_{d,sp}}\;\left(i,j,t\right) $$

The expression for the relaxation parameter *ω*_*sp*_ (indicated in Eq. (2)) can be deduced using the Enskog-Chapman procedure, and is given by [30]

5 $$ {\omega}_{sp}=\frac{2}{1+4{D}_{sp}^0\;\frac{\Delta t}{\Delta {x}^2}} $$

Due to the fact that the BGK model was formulated for non-crowded systems [19] *D*_*sp*_^0^ takes the value of the diffusion coefficient for diluted solutions. Here, *D*_*sp*_^0^ is considered independent of the position (*i,j*).

#### 2. Crowding-adaptation of LBM

According to Fick’s first law the diffusive flux of volumeless molecules (*J*) from one region to another is proportional to the gradient of the concentration. However, when the volume of the molecules is important and/or the solution is not considered diluted, *J* should be proportional to gradient of the activities [31].

Since the molecules in a defined system occupy a volume in space, not all the system’s volume is available to the centre of mass of a test molecule [6].

The activity *a* is a term that describes the number of molecules per available volume, while the concentration *C* is the number of molecules per total volume [6]. Both variables are related by Eq. (6), where the activity coefficient *γ* indicates deviations from an ideal solution.

6 $$ {a}_{sp}={\gamma}_{sp}\frac{C_{sp}}{C_{sp}^{st}} $$

Since *a*_*sp*_ is a dimensionless variable, for the purpose of dimensional consistency in this paper the standard concentration of the species *sp* (*C*_*sp*_^*st*^) is considered equal to 1 molecule per voxel, i.e., diluted solutions where there is no crowding influence. Hence, the *a*_*sp*_ value is not affected by *C*_*sp*_^*st*^, therefore Eq. (6) is simplified to *a*_*sp*_ = *γ*_*sp*_ *C*_*sp*_.

*γ* is defined as the ratio of the total volume (or area in 2D systems) divided by the available volume (or area in 2D), in other words, it is inversely proportional to the probability to find free space in the system.

From the above and for the lattice-system analysed here, the flux of the molecules (*J*_*sp*_) of the species *sp* in the *x* direction from voxel (*i,j*) to (*i,j* + 1) is defined in Eq. (7). The reverse flux is *J*_*sp*(*i*,*j* + 1 → *i*,*j*)_ = − *J*_*sp*(*i*,*j* → *i*,*j* + 1)_.

7 $$ {J}_{sp\left(i,j\to i,j+1\right)}=\frac{D_{sp}}{\Delta x}\left[{a}_{sp\left(i,j\to i,j+1\right)}\left(i,j,t\right)-{a}_{sp\left(i,j+1\to i,j\right)}\left(i,j+1,t\right)\right] $$

where the concentration involved in the term *a*_*sp*(*i*,*j* → *i*,*j* + 1)_ is proportional to the number of molecules (per voxel) trying to move from (*i,j*) to (*i,j* + 1) at time *t*, i.e., *F*_1,*sp*_^*LB*^(*i*, *j*, *t*), while *a*_*sp*(*i*,*j* + 1 → *i*,*j*)_ is proportional to *F*_3,*sp*_^*LB*^(*i*, *j* + 1, *t*).

Substituting Eq. (1) in Eq. (2), and using as concentration the values *F*_1,*sp*_^*LB*^(*i*, *j*, *t*) and *F*_3,*sp*_^*LB*^(*i*, *j* + 1, *t*) yields:

8 $$ {J}_{sp\left(i,j\to i,j+1\right)}=\frac{D_{sp}}{\Delta x}\left[{\gamma}_{sp}\left(i,j,t\right){F}_{1,sp}^{LB}\left(i,j,t\right)-{\gamma}_{sp}\left(i,j+1,t\right){F}_{3,sp}^{LB}\left(i,j+1,t\right)\right] $$

The use of a constant *D*_*sp*_ independent of the concentration could be questionable for crowded and inhomogeneous systems since the presence of background/crowder molecules hinders the movement of a test molecule.

Due to the steric effects, the presence of background molecules leads to a reduction of the available space where the test molecule can move due to Brownian motion. Therefore the probability to find free space next to the test molecule also decreases.

Considering that the rate of Brownian displacement (or diffusion) is a function of the work required to free the target space from background molecules (∆*W*), Muramatsu and Minton [32] proposed the relation

9 $$ \ln \left(\frac{D_{sp}}{D_{sp}^0}\right)=-\frac{\Delta W}{k_BT} $$

where *D*_*sp*_^0^ is the diffusion coefficient of species *sp* in diluted solutions. ∆*W* depends on the size and shape of the space required. The probability of observing the spontaneous formation of such free space as result of a fluctuation is [33, 34]

10 $$ {P}_{sp}={ \exp}^{-\frac{\Delta W}{k_BT}} $$

where *k*_*B*_ is the Boltzmann constant, and *T* is the temperature. Assuming a well-mixed system (or subsystem), the probability *P*_*sp*_ is defined as the available volume divided by the total volume, i.e., the inverse of the activity coefficient

11 $$ {P}_{sp}=\frac{1}{\gamma_{sp}} $$

Because the diffusion coefficient *D*_*sp*_ is a function of the available volume in the target voxel where the test molecule is trying to move in (and therefore dependent of the position (*i,j*)), *D*_*sp*_ cannot be factored as it is in Eq. (8), so

12 $$ {J}_{sp\left(i,j\to i,j+1\right)}=\frac{1}{\Delta x}\left[{\gamma}_{sp}\left(i,j,t\right){F}_{1,sp}^{LB}\left(i,j,t\right){D}_{sp}\left(i,j+1,t\right)-{\gamma}_{sp}\left(i,j+1,t\right){F}_{3,sp}^{LB}\left(i,j+1,t\right){D}_{sp}\left(i,j,t\right)\right] $$

Substituting Eq. (9) and (10) in Eq. (12), the flux *J*_*sp*(*i*,*j* → *i*,*j* + 1)_ can be rewritten as:

13 $$ {J}_{sp\left(i,j\to i,j+1\right)}=\frac{D_{sp}^0}{\Delta x}\left[{\gamma}_{sp}\left(i,j,t\right){F}_{1,sp}^{LB}\left(i,j,t\right){P}_{sp}\left(i,j+1,t\right)-{\gamma}_{sp}\left(i,j+1,t\right){F}_{3,sp}^{LB}\left(i,j+1,t\right){P}_{sp}\left(i,j,t\right)\right] $$

where *P*_*sp*_ is the probability of species *sp* to find available space in the target voxel. Eq. (13) looks like the Teorell formula for non-perfect systems [31].

In order to conserve the driving force, the corrected values *F*_*1,sp*_(*i,j,t*) and *F*_*3,sp*_(*i,j* + 1*,t*) should also be related to the flux *J*_*sp*(*i*,*j* → *i*,*j* + 1)_, so that

14 $$ {J}_{sp\left(i,j\to i,j+1\right)}=\frac{D_{{}_{sp}}^0}{\Delta x}\left[{\gamma}_{sp}\left(i,j,t\right){F}_{1,sp}\left(i,j,t\right)-{\gamma}_{sp}\left(i,j+1,t\right){F}_{3,sp}\left(i,j+1,t\right)\right] $$

Comparing Eq. (13) and Eq. (14), the new *F*_*1,sp*_(*i,j,t*) and *F*_*3,sp*_(*i,j* + 1*,t*) values are found as

15 $$ {F}_{1,sp}\left(i,j,t\right)={F}_{1,sp}^{LB}\left(i,j,t\right){P}_{sp}\left(i,j+1,t\right) $$

16 $$ {F}_{3,sp}\left(i,j+1,t\right)={F}_{3,sp}^{LB}\left(i,j+1,t\right){P}_{sp}\left(i,j,t\right) $$

Generalizing, the corrected values *F*_*d,sp*_ (*d* = 1,2,3,4) are calculated as

17 $$ {F}_{d,sp}\kern0.28em \left(i,j,t\right)={F}_{d,sp}^{LB}\kern0.28em \left(i,j,t\right)\kern0.28em {P}_{sp}\kern0.28em \left({i}_{next},{j}_{next},t\right)\kern.20em \mathrm{when}\kern0.2em d=1,2,3,4 $$

Or their equivalent:

18 $$ {F}_{d,sp}\left(i,j,t\right)=\frac{F_{d,sp}^{LB}\left(i,j,t\right)}{\gamma_{sp}\left({i}_{next},{j}_{next},t\right)} $$

The molecules that could not move into their corresponding target voxel (*i*_*next*_ *,j*_*next*_), i.e., the voxel next to (*i,j*) in the direction *d*, will remain in the current (*i,j*). Therefore in order to conserve mass *F*_0*,sp*_ becomes

19 $$ {F}_{0,sp}\;\left(i,j,t\right)=\frac{\rho_{sp}\;\left(i,j,t\right)}{m_{sp}}-{\displaystyle \sum_{d=1}^4{F}_{d,sp}\;\left(i,j,t\right)} $$

The activity coefficient *γ*_*sp*_ (*i,j,t*) is a function of the size and shape of the molecules present in the voxel/site (*i,j,t*).

In this paper, the Scale Particle Theory [33, 34] is used to approximate *γ*_*sp*_ in a mixture of (non-overlapping) hard disk-molecules (Eq. (20) and (21)) which could be of different radii *r* [31]. For this we assume that the volume and temperature of the subsystems/voxels are held constant. In order to simplify the notation, the position-time dependence (*i,j,t*) of the variables *γ*_*sp*_ and *S*_*x*_ has been dropped from Eq. (20) and (21).

20 $$ \ln {\gamma}_{sp}=- \ln \left(1-{S}_2\right)+\left[\frac{2{S}_1}{1-{S}_2}\right]{r}_{sp}+\left[\frac{S_0}{1-{S}_2}+\frac{S_1^2}{{\left(1-{S}_2\right)}^2}\right]{r}_{sp}^2 $$

where *S*_*x*_ (0 ≤ *x* ≤ 2) is given by

21 $$ {S}_x=\frac{\pi }{\Delta {x}^2}{\displaystyle \sum_{i=1}^l{\rho}_i}{\left({r}_i\right)}^x $$

Note that if all the particles simulated are considered to be point-like molecules (*r*_*i*_ → 0, where the space fraction occupied by the molecules tends to zero *S*_2_ → 0), and/or they are in a non-crowded system (${\displaystyle \sum_{i=1}^l{\rho}_i}\to 0$, hence *S*_*x*_ → 0), then ln(*γ*_*sp*_) → − ln(1) in Eq. (20) so that the probability *P*_*sp*_ (Eq. (11)) would be equal to 1, therefore *F*_*d,sp*_ becomes equal to *F*_*d*,*sp*_^*LB*^ (Eq. (17)).

The use of *P*_*sp*_ (or its equivalent *γ*_*sp*_^− 1^) in Eq. (17) restricts the maximum number of molecules in a voxel, and also avoids the exchange of positions between molecules. When two molecules moving in opposite directions are adjacent and have volume (or area in 2D systems), one acts as an obstacle for the other, so exchanging positions between them should be prohibited. In other words the displacement of a molecule is limited by the probability to find empty space *P*_*sp*_.

#### 3. Streaming step

The new *F*_*d ,sp*_ values are used in the streaming step in the same way as in LBM (Eq. (22) − (26) below). Then the scheme goes back to step 1 and the procedure is repeated until the simulation end time is reached.

22 $$ {F}_{1,sp}\left(i,j+1,t+\Delta t\right)={F}_{1,sp}\left(i,j,t\right) $$

23 $$ {F}_{2,sp}\left(i-1,j,t+\Delta t\right)={F}_{2,sp}\left(i,j,t\right) $$

24 $$ {F}_{3,sp}\left(i,j-1,t+\Delta t\right)={F}_{3,sp}\left(i,j,t\right) $$

25 $$ {F}_{4,sp}\left(i+1,j,t+\Delta t\right)={F}_{4,sp}\left(i,j,t\right) $$

26 $$ {F}_{0,sp}\left(i,j,t+\Delta t\right)={F}_{0,sp}\left(i,j,t\right) $$

Unlike (microscopic-) MC methods that track the movements of every single particle, (mesoscopic-) cLBM simulates the movement of collections of molecules across a (lattice) system. Hence, when a large number of molecules is considered and/or for long time simulations, cLBM simulations are computationally more efficient than MC methods. However, since each (cLBM-) voxel can fit more than one molecules and assuming that the voxel is well mixed, which is not necessarily true especially for large voxels, some discrepancies could be found between the diffusion results computed by cLBM and MC (in this paper we use kMC).

In order to validate the crowding-adaptation of LBM presented above, the simulation of a diffusion system was carried out by an on-lattice kMC algorithm [14, 15], whose results are used as our computational experiments. A brief description of the kMC algorithm is given below.

### Lattice Kinetic Monte Carlo (kMC) algorithm

In the on-lattice kMC [14] algorithm each site of the lattice can be occupied by at most one molecule. Each molecule can move to the one of the 4 neighbouring sites (top, bottom, right, or left), as long as they are free, i.e., there is no other molecule in the target site.

The basic idea of the procedure is the following:

At every time step

1. Identify the classes of species, i.e., the combinations of adjacent species that can react or diffuse (if there is an empty site next to a molecule).
2. The rates (including the diffusion rate) of all the possible events or processes are listed and their cumulative value is computed.
3. An event is probabilistically chosen, e.g., the next molecule to move and the site where it will take place, using a random number and the cumulative value of the rates listed in point 2.
4. The diffusion (or reaction) of the chosen molecule(s) takes place.
5. A variable time step value is also probabilistically estimated using another random number and the cumulative value of the rates.
6. The number of species and classes in a region and/or in the whole lattice is updated as well as the time.
7. Go back to point 1 until the end simulation time is reached.

See [14, 15] for detailed information of the algorithm and the corresponding equations for each step of the process.

MC algorithms have been widely used for the simulation of processes in crowded media [10–13, 16, 17]. The main difference between kMC and MC is that the former use a constant ∆*x* and a variable ∆*t*, while in MC both parameters are constant.

However for both cases, kMC and MC, a molecule only can move if the target (adjacent) site, which was randomly chosen is free/empty. Hence, the excluded volume restriction is straightforward satisfied in both kMC and MC. A comparison of the results of diffusion simulation obtained from these two algorithms is shown in the Additional file 1.

## Results and discussion

For the diffusion examples presented in this section the following assumptions have been made:

1. The system analysed consists of a square lattice of (1000 nm)^2^, divided in equal-sized voxels or sites of ∆*x* per side whose value is indicated in each example.
2. Each voxel is well-mixed.
3. The fluid phase is considered as a continuum and it is at rest.
4. The system has periodic boundaries.
5. The mass of all species are assumed equal to 1 g per molecule, therefore the value of *ρ*_*sp*_ is equal to the number of molecules, i.e., ${\displaystyle \sum_d{F}_{d,sp}}$. Notice that *m*_*sp*_ can take other values if it is required.

The methodologies LBM and cLBM were programed in MATLAB R2011a (The MathWorks, Natick, MA), while the lattice kMC was implemented in Fortran 90.

### Validation of cLBM: a lattice-model

**Fig. 2:**
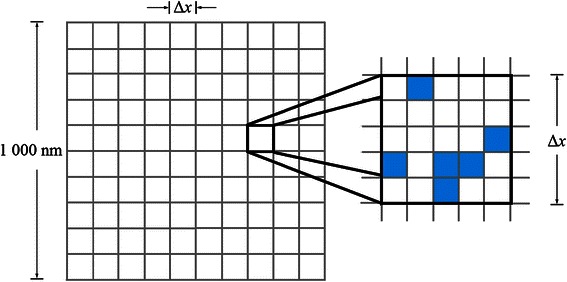
Lattice model of square uniform packing order. The main grid represents the cLBM/LBM voxels in which the system was divided. The molecules are represented by dashed squares (in the same way as in kMC algorithm)

For validation purposes, we consider a lattice model (Fig. 2) for the diffusion of molecules here represented by non-rotating squares of (10 nm)^2^ which have a square uniform packing order. Taking into account that the system’ size is (1000 nm)^2^ the maximum number of molecules that can fit inside is 10,000.

This type of system is consistent with the ones commonly used in on-lattice kMC simulations where a molecule can move to one of its neighbouring sites at every time step.

According to the ∆*x* value used in the cLBM simulations, for comparison purposes, the average results obtained (after 1000 repetitions) at every time step ∆*t* by kMC were coarse grained in lattice regions equivalent to voxels of side length equal to ∆*x*.

Due to the square uniform packing order of the same size molecules in this lattice model the activity coefficient *γ*_*sp*_ (which is required in cLBM) given by Eq. (20) and (21) can be simplified to

27 $$ \ln {\gamma}_{sp}=- \ln \left(1-{S}_2\right) $$

28 $$ {S}_2=\frac{1}{\Delta {x}^2}{\displaystyle \sum_{i=1}^m{\rho}_i}{A}_i $$

where *A*_*i*_ is the area of a molecule of species *i*.

**Fig. 3:**
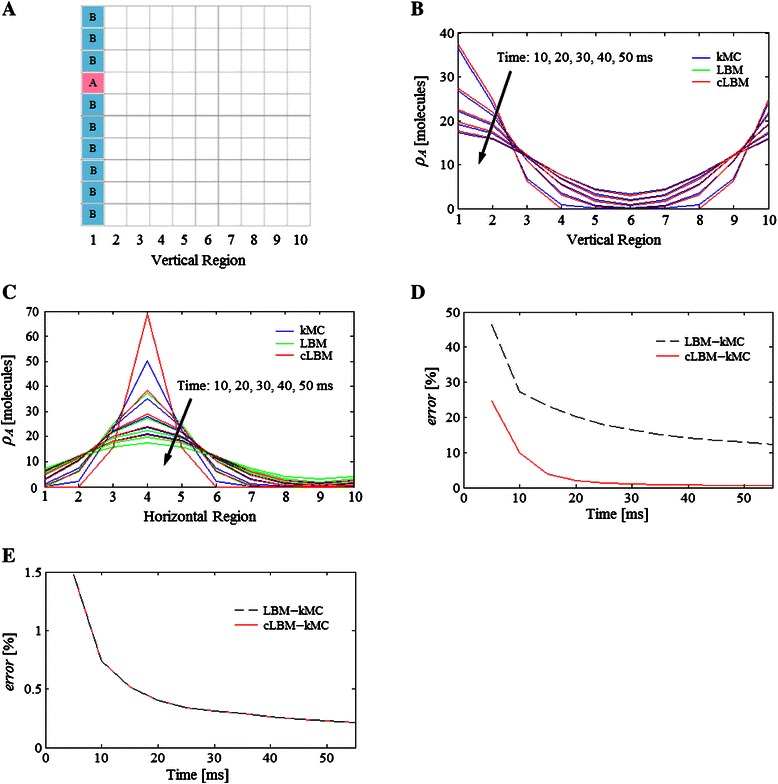
Diffusion example 1. **a** Initial location of a system composed by of two types of molecules. Diffusion profile of the test molecules A predicted by LBM, cLBM, and kMC in (**b**) the horizontal direction and (**c**) vertical direction at times 10, 20, 30, 40 and 50 ms. Notice that in the horizontal direction (**b**) identical profiles are predicted by LBM (green line) and cLBM (red line), therefore the red line is on top of the green one. **d** Relative error of the distribution of molecules A estimated by cLBM − kMC and LBM − kMC. **e** Relative error predicted assuming that all the molecules (**a** and **b**) are of the same type. The parameters used in the simulation are indicated in Table 1

The diffusion of two types of molecules has been simulated using the parameters shown in Example 1 of Table 1. For this, the lattice is divided into 10 vertical regions, where the voxels (of ∆*x* = 100 nm) of the first column of the lattice have been filled with 100 molecules of type A and B as indicated in the Fig. 3a.

**Table 1:** Parameters used in the diffusion examples

Example 1	Example 2	Example 3	Example 4	Example 5
Δ*x* = 100	Δ*x* = 50	Δ*x* = 50	Δ*x* = 50	Δ*x* = 50
Δ*t* = 5	Δ*t* = 0.625	Δ*t* = 0.625	Δ*t* = 0.625	Δ*t* = 0.625
*D*_*A*_ = 500	*D*_*A*_ = 1 000	*D*_*tracer*_ = 1 000	*D*_*tracer*_ = 1 000	*D*_*tracer*_ = 1 000
*A*_*A*_ = 100	*A*_*A*_ = 100	*A*_*tracer*_ = 100	*r*_*tracer*_ = 2, or 1.5, or 1, or 0	*r*_*tracer*_ = 1.5
*C*_*A*_ = 100	*C*_*A*_ = 1 000	*C*_*tracer*_ = 100	*C*_*tracer*_ = 796	*C*_*tracer*_ = 1 414
*D*_*B*_ = 500	*D*_*B*_ = 1 050	*D*_*crowder*_ = 1 000	*D*_*crowder1*_ = 1 050	*D*_*crowder*_ = 1 000
*A*_*B*_ = 100	*A*_*B*_ = 100	*A*_*crowder*_ = 100	*r*_*crowder1*_ = 1.9	*r*_*crowder*_ = 2
*C*_*B*_ = 900	*C*_*B*_ = 1 000	*C*_*crowder*_ = 1 000 or	*C*_*crowder1*_ = 6 172	*C*_*crowder*_ = 23 873 or
	*D*_*C*_ = 1 100	*C*_*crowder*_ = 2 000 or	*D*_*crowder2*_ = 1 100	*r*_*crowder*_ = 1.5
	*A*_*C*_ = 100	*C*_*crowder*_ = 3 000 or	*r*_*crowder2*_ = 1.8	*C*_*crowder*_ = 42 441 or
	*C*_*C*_ = 1 000	*C*_*crowder*_ = 4 000	*C*_*crowder2*_ = 9 824	*r*_*crowder*_ = 1
			*D*_*crowder3*_ = 1 200	*C*_*crowder*_ = 95 493
			*r*_*crowder3*_ = 1.6	
			*C*_*crowder3*_ = 6 217	
			*D*_*crowder4*_ = 1 250	
			*r*_*crowder4*_ = 1.5	
			*C*_*crowder4*_ = 11 318	

The parameters indicated above are expressed in units: Δ*x* [=] nm, Δ*t* [=] ms, *D*_*sp*_ [=] nm^2^ ms^−1^, *A*_*sp*_ [=] nm^2^, *C*_*sp*_ [=] molecules per (1 000 nm)^2^

The order of magnitude of diffusion coefficients used agrees with that reported by Elowitz et al. [41]

Once the molecules begin to diffuse, it is possible to compare their movement summing the number of molecules A (used as tracer molecule) in each vertical region of the lattice at different times. Figure 3b shows that LBM and cLBM predict the same diffusion profile, which in turn is close to the results estimated by kMC. This is because there are no obstacles in the horizontal direction and therefore cLBM results are the same as the ones from LBM. The same happens when the volume (or area in 2D) of the molecules are zero (data not shown).

If we compare the molecules’ movement in the vertical direction (summing the number of molecules A but now in each horizontal region), then it is possible to see differences between the diffusion profile of cLBM and LBM (Fig. 3c). Results show that the system evolves faster, i.e., LMB computes that more molecules move or diffuse to their neighbouring voxels, than cLBM. Hence, the profile predicted by cLBM in the vertical direction (where the obstacles are initially located) is closer to the one obtained by kMC than that predicted by LBM.

The relative error of the results obtained by cLBM and/or LBM compared with kMC is calculated as the Frobenius norm of the difference of the matrixes containing the number of molecules *sp* in each voxel predicted by kMC (**ρ**_**kMC**_) and cLBM (**ρ**_**cLBM**_), or by kMC and LBM (**ρ**_**LBM**_), divided by the total number of molecules *sp* simulated, i.e.,

29 $$ erro{r}_{sp}=\frac{norm\left({\boldsymbol{\uprho}}_{sp,\mathbf{k}\mathbf{M}\mathbf{C}}-{\boldsymbol{\uprho}}_{sp,\mathbf{cLBM}}\right)}{{\displaystyle \sum_i^m{\displaystyle \sum_j^m{\rho}_{sp,kMC}\left(i,j\right)}}}100=\frac{\sqrt{{\displaystyle \sum_i^m{\displaystyle \sum_j^m{\left({\rho}_{sp,kMC}\left(i,j\right)-{\rho}_{sp, cLBM}\left(i,j\right)\right)}^2}}}}{{\displaystyle \sum_i^m{\displaystyle \sum_j^m{\rho}_{sp,kMC}\left(i,j\right)}}}100 $$

Since *m*_*sp*_ of all *sp* is assumed to be equal to 1 g per molecule, we keep the term *ρ*_*sp*_ to represent the number of molecules in the system.

A comparison of the *error*(**ρ**_**kMC**_ − **ρ**_**cLBM**_) and *error*(**ρ**_**kMC**_ − **ρ**_**LBM**_) for the diffusion of species A in the Example 1 indicates that cLBM predictions are more accurate than those of LBM (Fig. 3d). For example, the relative error estimated by cLBM at time 30 ms is *error*_*A*_ = 0.93 % (which indicates that from 100 molecules A simulated, cLBM predicts that 0.93 molecules are allocated in different voxel’s positions than those predicted by kMC, in other words there are deviations in the distribution of 0.93 molecules A from the 100 simulated in this example), while the error from LBM amounts to *error*_*A*_ = 16.39 %.

This is because LBM considers point-like molecules so that they will always find enough space to fit in the target voxel, causing LBM to over-predict diffusion in the vertical direction.

Nevertheless, if we assume that both molecules A and B are of the same type then the relative error between kMC and LBM decreases, becoming equal to that between kMC and cLBM (Fig. 3e). This means that LBM is a good alternative for quick simulations of one type of molecules, but when two or more species are present then it can over predict the system’s degree of mixing.

**Fig. 4:**
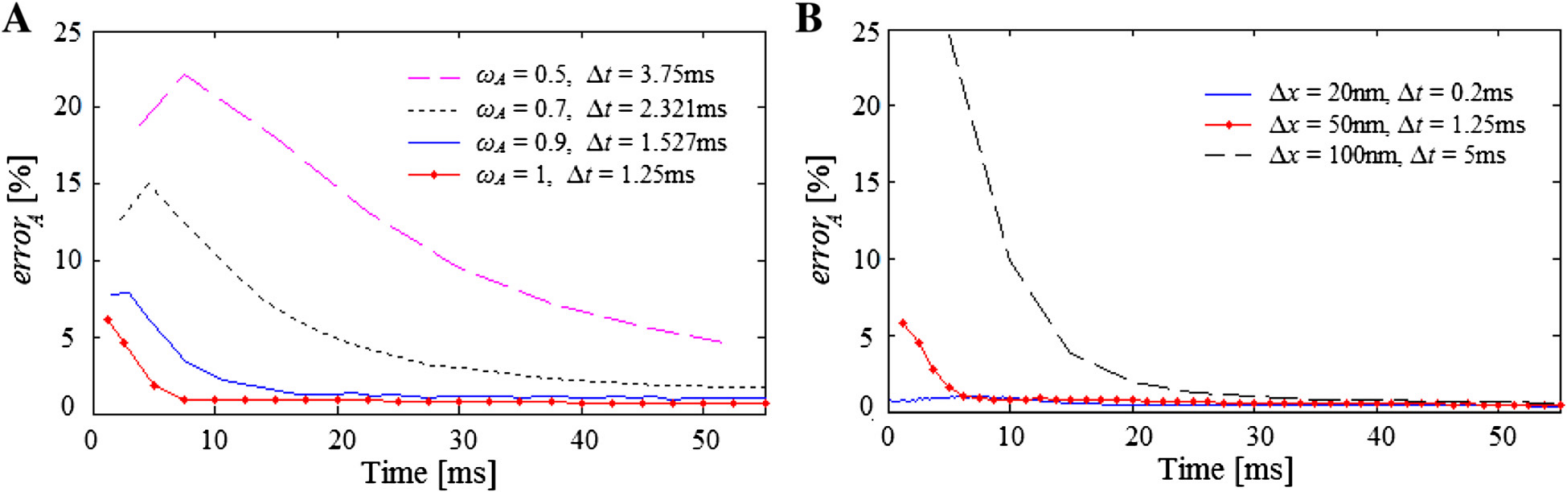
Relative error of the molecules distribution estimated by cLBM − kMC for Example 1. **a** Different relaxation parameter *ω*_*A*_ were tested but maintaining constant ∆*x* = 50 nm. **b** Different ∆*x* and ∆*t* values were tested maintaining *ω*_*A*_ = 1

As was pointed out by Li et al. [35], the relaxation parameter value affects the accuracy of LBM. According to Eq. (5) and the chosen parameters ∆*x* and ∆*t* in Example 1, both *ω*_*A*_ and *ω*_*B*_ were estimated equal to one. In order to show the dependency between the *error*_*A*_ and *ω*_*A*_, we tested different ∆*t* values, maintaining constant ∆*x* = 50 nm, which corresponds to *ω*_*A*_ ranging from 0.5 to 1. The results indicate that *ω*_*A*_ = 1 gives the lowest *error*_*A*_ (Fig. 4a), therefore in the following we use ∆*x* and ∆*t* values that allow setting *ω*_*sp*_ close to one.

From Eq. (5) when *ω*_*sp*_ = 1, then the diffusion coefficient ${D}_{sp}=\frac{\Delta {x}^2}{4\Delta t}$, is equal to the stability limit, $\Delta t\le \frac{\Delta {x}^2}{4{D}_{sp}}$, given by the finite difference (FD) approximation of the diffusion equation $\frac{\partial {\rho}_{sp}}{\partial t}={D}_{sp}{\nabla}^2{\rho}_{sp}$. Under this condition (*ω*_*sp*_ = 1), LBM estimates ${F}_{d,sp}^{LB}\;\left({i}_{next},{j}_{next},t+\Delta t\right)=\frac{\rho_{sp}\left(i,j,t\right)}{m_{sp}}{w}_d$ (see Eq. (1)–(3)), that substituting in Eq. (4) gives the same equation obtained by FD at the stability limit [19], i.e.,

30 $$ {\rho}_{sp}\left({i}_{next},{j}_{next},t+\varDelta t\right)=\frac{\rho_{sp}\left({i}_{next}-1,{j}_{next},t\right)+{\rho}_{sp}\left({i}_{next}+1,{j}_{next},t\right)+{\rho}_{sp}\left({i}_{next},{j}_{next}-1,t\right)+{\rho}_{sp}\left({i}_{next},{j}_{next}+1,t\right)}{4} $$

Moreover, since cLBM is formulated as an explicit method that requires information of the neighbouring voxels at time *t* to estimate *ρ*_*sp*_ of a voxel (*i,j*) for the next time *t* + ∆*t*, decreasing ∆*t* also diminishes the error predicted between kMC and cLBM for Example 1 as shown in Fig. 4b, where 3 different sets of coefficient ∆*t* and the corresponding ∆*x* that keeps constant *ω*_*sp*_ = 1 were tested.

In all cases, the LB methods required *s* time steps of length ∆*t*, before reaching a quasi-stable error. Therefore the smaller the chosen ∆*t* (and the corresponding ∆*x*) the faster the system reached that state. Since good results were obtained with ∆*t* = 1.25 ms and ∆*x* = 50 nm (where a voxel allows to fit a maximum of 25 molecules) compared with the more accurate but slower ∆*t* = 0.2 ms and ∆*x* = 20 nm, in the following we use ∆*x* = 50 nm.

**Fig. 5:**
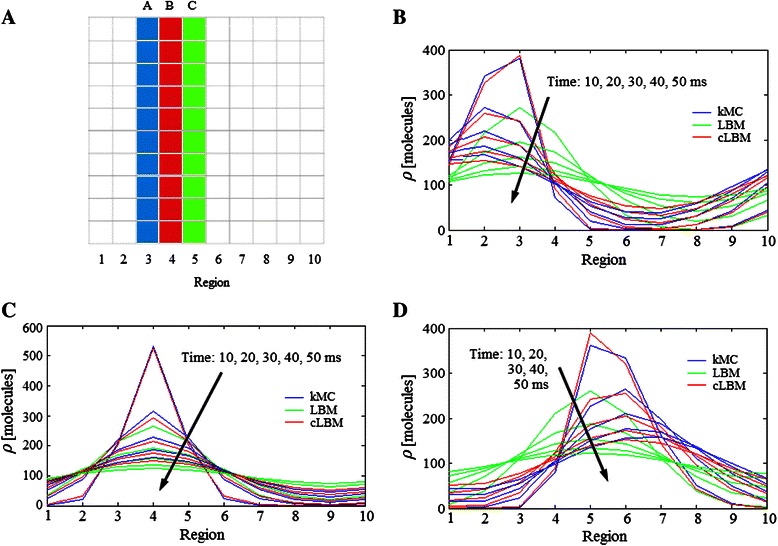
Diffusion example 2. **a** Initial conditions of the diffusion of three types of molecules. Diffusion profile of the species (**b**) A, (**c**) B, and (**d**) C predicted by LBM, cLBM, and kMC at times 10, 20, 30, 40, 50 ms

In the previous example the crowding conditions are in the vertical direction so that reduced displacement of species A is in this direction (Fig. 3c). Continuing the analysis of the crowding effect on the molecules’ displacement, a second example is proposed for the diffusion of three types of molecules initially allocated in the region indicated in Fig. 5a (the parameters are shown in Example 2 of Table 1).

For the purpose of this example, a region consists of two columns of 20 voxels with ∆*x* = 50 nm, where each voxel of region 3 (Fig. 5a) is filled with 25 molecules of species A. Region 4 and 5 are filled with equal numbers of species B and C, respectively. The results of the diffusion of species A show that cLBM predicts the same behaviour as kMC as can be seen in Fig. 5b.

On the other hand, LBM predicts a symmetric movement of A despite the fact that species B acts as an obstacle in its way (Fig. 5b). The same symmetric profile is obtained for species B and C (Fig. 5c and d).

**Fig. 6:**
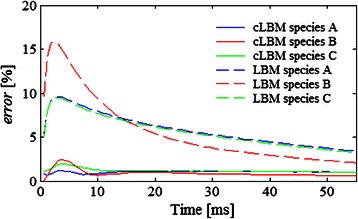
Relative error of the molecules distribution estimated by kMC − cLBM and kMC − LBM for Example 2

A comparison of the relative error between kMC − cLBM and kMC − LBM is shown in Fig. 6. As was also observed in Example 1, the error computed with the cLBM results is smaller than that computed through the LBM results. In fact, at time 30 ms the errors given by cLBM (*error*_*A*_ = 1.103 %, *error*_*B*_ = 0.833 %, *error*_*C*_ = 1.172 %) represent deviations in no more than 2 % of the total number of molecules simulated for each species.

Additionally, a comparison of the mean squared displacement (MSD) of a tracer molecule, i.e., the displacement from one voxel to another, was carried out under different crowding conditions (the parameters are given in Example 3 of Table 1). The total number of tracer molecules represents 1 % of the lattice area of the 2D system simulated by cLBM and LBM.

**Fig. 7:**
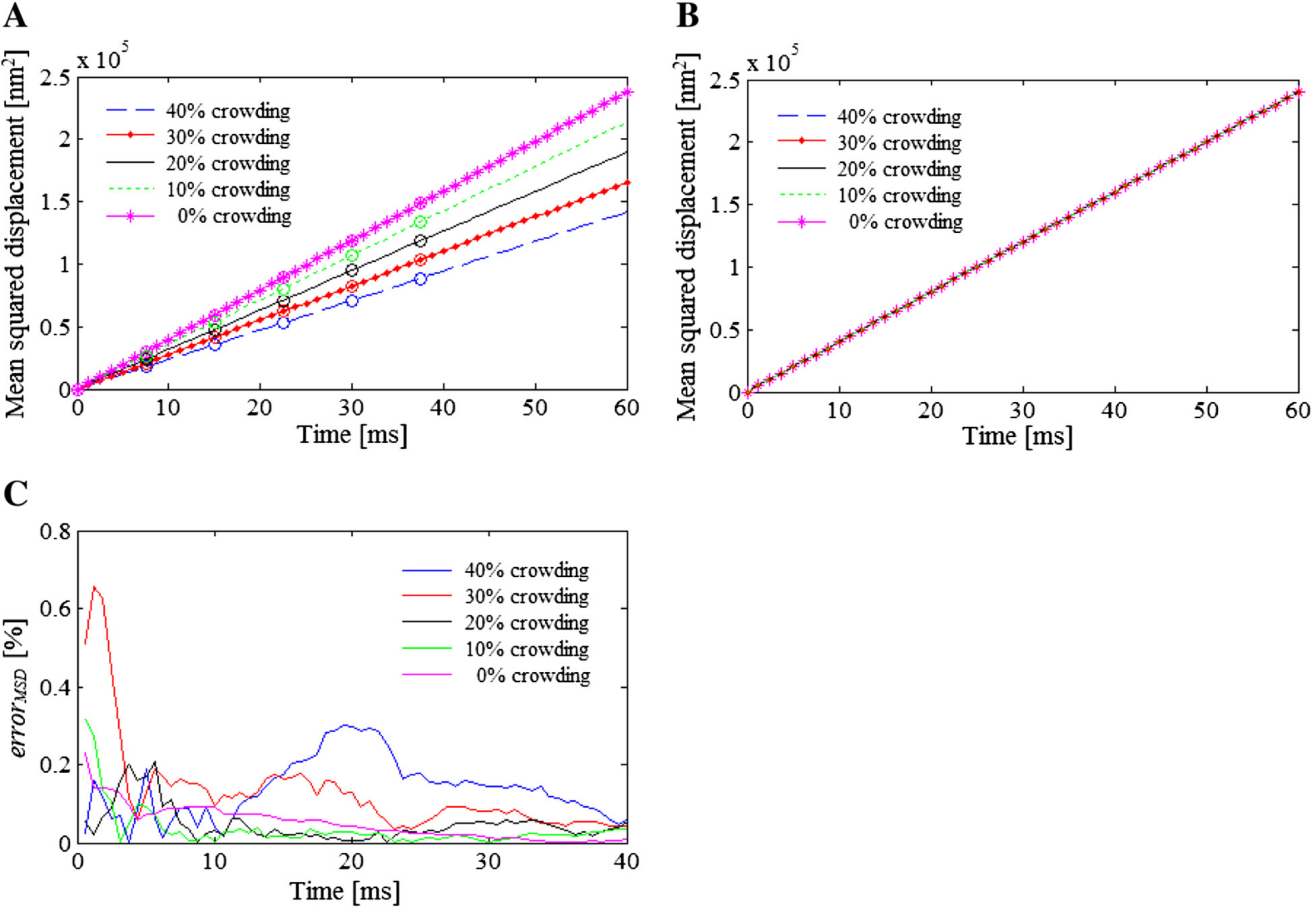
Diffusion example 3. Mean squared displacement of tracer molecules representing 1 % of the lattice area at different concentration of crowder molecules: 0, 10, 20, 30, and 40 % of the lattice area, predicted by (**a**) cLBM and (**b**) LBM. The circles in (**a**) represent the MSD computed by kMC for each condition tested. **c** Relative error of the tracer’s MSD estimated by cLBM − kMC. The parameters used in the simulation are indicated in Table 1

The results show (Fig. 7a and b) that only cLBM is sensitive to increments in the concentration of the background/crowder molecules, which is reflected in a reduction of the displacement of the tracer particle.

The comparison of the MSD computed by cLBM (lines and dashed lines in Fig. 7a) and kMC (circles in Fig. 7a) reveals a very good agreement between both methods. The error estimated between *MSD*_*cLBM*_ and *MSD*_*kMC*_, $erro{r}_{MSD}(t)=\frac{norm\left(MS{D}_{kMC}(t)-MS{D}_{cLBM}(t)\right)}{MS{D}_{kMC}(t)}100$, was found to be lower than 0.7 % for all the crowding conditions tested (Fig. 7c). These small differences in the tracer’s displacement may be due to the fact that LB methods are unable to identify and quantify the motion of the molecules that remain in the same voxel at time *t* + Δ*t*, i.e., LB methods only quantify the “effective” displacement made when the molecules pass from one to another voxel, but not how many movements have to be executed before entering to the next voxel as is the case with lattice kMC.

Despite the fact that kMC and cLBM were implemented in different computing languages (Fortran and Matlab, respectively, using an Intel Xeon 5160, CPU 3.00 Hz processor) a comparison of the execution time (CPU time) for the diffusion Example 1 (*t*_*kMC*_ = 315 s per run simulation or repetition vs *t*_*CLBM*_ = 7.13 s), and Example 2 (*t*_*kMC*_ = 441 s per repetition vs *t*_*CLBM*_ = 18.31 s) reveals the potential use of cLBM for faster simulations of larger systems. The total time required by kMC depends on the number of repetitions performed. The minimum error estimated between kMC and cLBM (Eq. (29)) for Example 1 after different kMC simulation repetitions was 1.43 % (50 repetitions ~ *t*_*kMC*_ = 4.37 hr), 1.32 % (100 repetitions ~ *t*_*kMC*_ = 8.75 hr), 0.71 % (500 repetitions ~ *t*_*kMC*_ = 43.75 hr), and 0.51 % (1000 repetitions ~ *t*_*kMC*_ = 87.5 hr). While in Example 2 the minimum error computed was 0.89 % (50 repetitions ~ *t*_*kMC*_ = 6.12 hr), 0.871 % (100 repetitions ~ *t*_*kMC*_ = 12.25 hr), 0.61 % (500 repetitions ~ *t*_*kMC*_ = 61.25 hr), and 0.54 % (1000 repetitions ~ *t*_*kMC*_ = 122.5 hr).

Certainly, the speed of cLBM-simulations depends on the time simulated and the chosen ∆*t*, but also other factors can affect its execution time, e.g., the number of different species analysed and the number of voxels in which the system is divided (i.e., the chosen ∆*x*). This is because *F*_*d,sp*_(*i*,*j*,*t*) is estimated for each species *sp* at every voxel position.

### Different size molecules in cLBM

Up to this point we have analysed a lattice-model were only molecules of the same size can be considered, however as was pointed out by Vilaseca et al. [10, 11], the size (and also the shape) of the molecules could be an important parameter in their movement or diffusion.

In order to study the influence of the size of the molecules on the diffusion process we simulate the motion of a tracer molecule in a 2D crowding system composed by 5 types of background particles (all of them of circular shape with different radii and concentrations) which are randomly located and all together occupy 30 % of the total lattice space (the corresponding parameters are given in Example 4 of Table 1).

According to the SPT assumption of a well-mixed voxel, here the molecules can be anywhere unlike the previous lattice model where only a square uniform packing order is allowed.

**Fig. 8:**
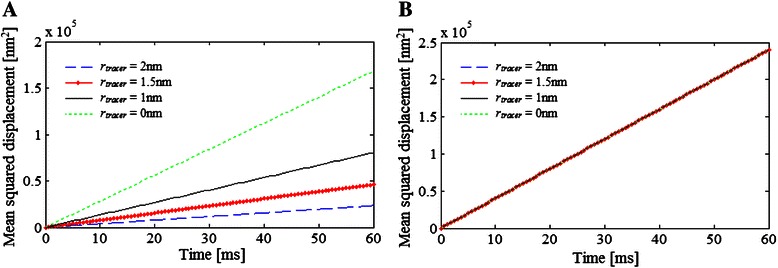
Diffusion example 4. Mean squared displacement estimated by (**a**) cLBM and (**b**) LBM for hard-disk tracer molecules of different radii: 2, or 1.5, or 1 or 0 nm, with total concentration equal to 796 molecules per (1000 nm)^2^, in a crowded media composed by 4 types of molecules which together occupied 30 % of the lattice area. The parameters used in the simulation are indicated in Table 1

Figure 8a shows the displacement across the lattice predicted by cLBM for a tracer molecule of different radius magnitude whose total concentration (i.e., the total number of molecules in the lattice) is 796 molecules. As it can be seen (Fig. 8) more movements from one voxel to another were detected when a small radius is assumed. This is because the probability of the tracer species to move to the target voxel increases inasmuch as the second and third term of Eq. (20) disappear when *r*_*tracer*_ = 0 nm. In other words, the available space for point-like molecules equals the free space in the target voxel, i.e., the total area not occupied by other molecules represented by the term 1 − *S*_2_ in Eq. (20).

Moreover, the comparison of the displacement of the tracer molecule with *r*_*tracer*_ = 0 nm obtained by cLBM (broken pink line in Fig. 8a) and that estimated by the classical LBM for the same Example 4 (broken pink line in Fig. 8b) indicates that even if the metabolites are considered as point-like particles they are still affected by the molecular crowding conditions, unless all the molecules simulated in the system are also point-like molecules.

Finally, the effect of the size of the crowder molecules on diffusion was investigated. For this, 3 different diffusion simulations of a system composed of tracer molecules of *r*_*tracer*_ = 1.5 nm which occupies 1 % of the lattice area, and crowder molecules having different radii: 2, 1.5, and 1 nm, representing 30 % of the total lattice space were performed.

**Fig. 9:**
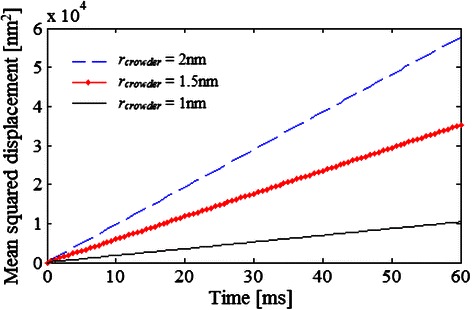
Diffusion example 5. Mean squared displacement of a tracer molecule in 3 different systems where the 30 % of the total lattice space is occupied by the same type of crowder molecules, but having different radii: 23 873 molecules of *r*_*crowder*_ = 2 nm, or 42 441 molecules of *r*_*crowder*_ = 1.5 nm, or 95 493 molecules of *r*_*crowder*_ = 1 nm). The radii and concentration of the tracer are 1.5 nm and 1 414 molecules, respectively. The parameters used in the simulation are indicated in Table 1

Since the area covered by the crowder is kept constant despite the fact that the radius is modified, more particles of *r*_*crowder*_ = 1 nm are simulated compared with those used if *r*_*crowder*_ is 1.5 or 2 nm. A comparison of the mean squared displacement of a tracer molecule (Fig. 9) under such conditions indicates that the particles’ motion decreases when the number of crowder molecules increases.

This suggests that not only the space fraction occupied by crowders is important but also the way it is covered, i.e., the size and number of molecules. Similar behaviour was found by Vilaseca et al. [10] for the diffusion simulation of molecules having different sizes using a Monte Carlo algorithm.

The (2D-) cLBM methodology presented in this paper is useful to simulate the diffusion in systems such as cell membranes. Furthermore, cLBM can be easily extended for diffusion simulations in three dimensions. Following the same reasoning for the estimation of the corrected value *F*_*d,sp*_ (Eq. (17)) there are only small differences between the equations used for 3D and those for 2D. For example the variables *F*_*d*,*sp*_^* LB*^, *F*_*d,sp*_, *ρ*_*sp*_, Ω_*d*,*sp*_^*diff*^, and *P*_*sp*_ will depend on the position (*i*,*j*,*k*), where the index *k* denotes the z-coordinate of the space. Besides, in the calculation of *ω*_*sp*_ (Eq. (5)) the coefficient 4 used in the second term of the denominator would change to the value 6, see [30] for the deduction of the relaxation parameter equation in 3D. Finally, the 2D SPT equation used to estimate *γ*_*sp*_ (Eq. 20) would also change to consider spherical molecules in 3D systems, see [33, 34] for more details.

Therefore, it is expected that 3D-cLBM will maintain the computational efficiency of 2D-cLBM. However, since the number of voxels will increase due to the dimensionality of the system, the time required to simulate the motion of molecules will increase accordingly.

On the other hand, reactions have been simulated by the classical LBM [35–38] through the law of mass action, which assumes that the reaction rate is proportional to the concentration of the reactants. However, more general speaking the reaction rate is proportional to the activity of the reactants (Eq. 6).

Considering the above, cLBM can be also extended to simulate reaction–diffusion systems in crowded media. For this, the reaction process takes place at every time step and in every voxel. The evolution (consumption/production) of the species in a well-mixed voxel is a function of the activity of the reactants in the voxel, i.e., the species’ concentration (or number of molecules per voxel equivalent to *ρ*_*sp*_) and *γ*_*sp*_ estimated by SPT.

Since the reaction processes have to be evaluated at every time step and in every voxel, an increase in the execution time is expected for the simulation reaction–diffusion systems proportional to the parameters ∆*x* and ∆*t*. Therefore strategies and simplifications will be required to optimise the simulation run time.

When the difference between the diffusion and reaction rates is important, strategies like the time splitting method [30] can be adopted into LBM and cLBM methods. For example, in the scheme DRD splitting the time step ∆*t* is divided in two ∆*t =* ∆*t*_1_ + ∆*t*_2_. First, the diffusion is carried out during ∆*t*_1_, then the reactions take place using the number of molecules present in the voxel after time ∆*t*_1_. Finally, the updated number of molecules (counted after the reactions took place) are allowed to diffuse during time ∆*t*_2_.

This paper focuses on the diffusion of macromolecules. However, when the simulation of a mixture of metabolites and macromolecules (where the diffusion coefficients could be of different order of magnitude) is required, then some strategies are needed to balance the computational cost with accuracy of cLBM. For example, the use of a very small ∆*x* will increase the number of voxels and therefore the computation effort required for the simulation. The use of the time splitting method (or similar strategies) will help in the simulation of diffusion of a mixture of metabolites and macromolecules. In this way cLBM may be extended for the reaction–diffusion simulation in more realistic (and crowded) scenarios.

## Conclusions

The LBM predicts with great accuracy the diffusion of particles under ideal conditions, i.e., considering point-like molecules, and/or non-crowding systems, and/or when only one type of molecules is simulated. However, if conditions change, for example, a system involving more than two species at crowding conditions, LBM predictions increasingly deviate from our on-lattice kMC-based computational experiment.

Although small discrepancies were found between the cLBM and kMC results (differences that were expected due to the level of detail inherent in each method), the proposed crowding adaptation of LBM is able to predict the same behaviour in the species diffusion profile. This suggests that the coupled SPT-LBM can be considered as a computational alternative for fast simulations of diffusion systems with a large number of molecules of different size and/or for long times, under crowding conditions at a fraction of the computational cost compared to a molecular (microscopic) method such as kMC.

Nevertheless, as in other mesoscopic methods, the saving in the execution time is accompanied by a reduction in the information that cLBM can provide compared with that obtained from a microscopic method. For example, the quantification of the total displacement of the molecules on time for the parameters estimation of anomalous diffusion [10, 11].

The accuracy of cLBM is influenced by the chosen voxel size ∆*x* and time increment ∆*t*, both related by the relaxation parameter *ω*_*sp*_. It was found that the use of *ω*_*sp*_ values close to one gives better results than any other in the range 0 ≤ *ω*_*sp*_ ≤ 1. Moreover, cLBM being an explicit method, small values of ∆*t* (maintaining *ω*_*sp*_ = 1, which involves reducing ∆*x*) also reduce the error between the proposed methodology (cLBM) and kMC.

Other factors that can affect the accuracy of cLBM is the area (or volume in 3D) fraction occupied by molecules, and the presence of immobile species. In particular, SPT (used in cLBM to estimate the probability to find available space *P*_*sp*_) works well for low to moderate area (or volume in 3D) fraction occupied by molecules [39, 40]. At high area (or volume) fraction occupied by molecules or when immobile particles are considered, the spatial distribution of the molecules inside a voxel could form free space “pockets” which will be not available for the incoming molecules from neighbouring voxels but that SPT takes into account in the calculation of *P*_*sp*_. This would lead to an overestimation of the molecules’ diffusion.

Regarding the influence of the size of the particles on the diffusion process, a reduction in the mean squared displacement of a tracer molecule when its size is increased was observed, as well as when the size of the crowders is decreased (but maintaining constant the lattice fraction occupied by them). Hence, the incorporation of small molecules, e.g., metabolites, in the simulation system can affect the diffusion profile predicted for macromolecules.

Even though cLBM requires the species’ concentration of the neighbour voxels at time *t* to compute the results at *t* + ∆*t*, therefore the LBM’s local feature is lost, the correction for the crowding effects is external to the estimation of LBM distributions, i.e., *F*_*d*,*sp*_^* LB*^, so that alternative LBM schemes can potentially be implemented within cLBM, e.g., for the simulation of reaction–diffusion systems.

### Nomenclature

*a*_*sp*_  Activity of the species *sp* [dimensionless].

*A*_*sp*_ Area of a molecule of the species *sp* [nm^2^].

*C*_*sp*_ Concentration of the species *sp* [molecules nm^−2^].

*C*_*sp*_^*st*^ Standard concentration of the species *sp* [molecules nm^−2^].

*d*    Direction chosen by the molecules to jump to neighboring voxel [dimensionless].

*D*_*sp*_ Diffusion coefficient [nm^2^ ms^−1^].

*D*_*sp*_^0^ Diffusion coefficient in dilute solutions [nm^2^ ms^−1^].

*error*_*sp*_  Relative error of molecules’ distribution predicted by kMC − cLBM or kMC − cLBM of the species *sp* [%].

*error*_*MSD*_Relative error of tracer’s MSD predicted by kMC − cLBM [%].

*F*_*d,sp*_ Distribution function of the species *sp* in the direction *d* predicted by cLBM [molecules per voxel].

*F*_*d*,*sp*_^*eq*^Equilibrium distribution function of the species *sp* in the direction *d* [molecules per voxel].

*F*_*d*,*sp*_^*LB*^Distribution function of the species *sp* in the direction *d* predicted by LBM [molecules per voxel].

*i*  Index that identify the position of a voxel [dimensionless].

*j* Index that identify the position of a voxel [dimensionless].

*J*_*sp*_ Diffusive flux of molecules *sp* in 2D [molecules nm^−1^ ms^−1^].

*m*_*sp*_ Mass of a molecule *sp* [g molecule^−1^].

*next* Subscript that indicates the target voxel where the molecules will move in the *t +* Δ*t* [dimensionless].

*P*_*sp*_ Probability to find available space for species *sp* in the target voxel [dimensionless].

*r*_*sp*_ Radii of the species *sp* [nm].

*k*_*B*_ Boltzmann constant equivalent to 1.3806 × 10^−23^ [J K^−1^].

*sp* Index that identify the molecule species *sp* [dimensionless].

*t* Time [s].

*T* Temperature of the medium [K].

*w*_*d*_Weight factor for the calculation of the equilibrium function [dimensionless].

Δ*t* Time increment [s].

∆*W*Work required to free the target space from background molecules [J molecule^−1^].

Δ*x*Size of the voxel in which the lattice is divided [nm].

*γ*_*sp*_Activity coefficient of a molecule *sp* [dimensionless].

Ω_*d*,*sp*_^*diff*^ Non-reactive collision term [molecules per voxel].

*ω*_*sp*_ Relaxation parameter [dimensionless].

*ρ*_*sp*_ Macroscopic density of species *sp* in a voxel [g per voxel].

**ρ**_*sp*_ Matrix with the macroscopic density of species *sp* in all voxels of the lattice [g voxel].

## Additional file

Additional file 1:
**A Lattice-Boltzmann scheme for the simulation of diffusion in intracellular crowded systems.** (PDF 363 kb)

## Abbreviations

LBM: Lattice Boltzmann Method
cLBM: crowding adaptation to Lattice Boltzmann Method
kMC: kinetic Monte Carlo algorithm
MC: Monte Carlo algorithm
