# Supplementary material for: A Lattice-Boltzmann scheme for the simulation of diffusion in intracellular crowded systems
Source: BMC Bioinformatics. 2015 Nov 3;16:353. doi: 10.1186/s12859-015-0769-8 (PMC4632338; doi:10.1186/s12859-015-0769-8)
Supplement: Additional file 1: — A Lattice-Boltzmann scheme for the simulation of diffusion in intracellular crowded systems. (PDF 363 kb) [file 12859_2015_769_MOESM1_ESM.pdf]

Additional file 1. A Lattice-Boltzmann scheme for the simulation of diffusion in intracellular crowded systems

Monte Carlo (MC) algorithm represents a powerful tool for the simulation of molecules' diffusion in crowded media. Due to its simplicity it has been widely used to simulate (reaction-) diffusion systems [1–6]. The kinetic Monte Carlo (kMC) algorithm is a MC method used in the simulation of surface reaction and diffusion systems [7–9].

The molecules' motion in a lattice (using a step size Δx) can be followed by both MC and kMC methods. One of the difference between them is that while in MC all the molecules try to move to the next site during a constant Δt, in kMC one event, i.e. the diffusion of one molecule, is (probabilistically) chosen along with its corresponding (variable) Δt, i.e. the time required for the displacement of that molecule, which depends on the diffusion coefficient (or reaction rate) of all the processes that are waiting to take place. Therefore, kMC requires as inputs the rate of all the processes to be simulated.

In both kMC and MC, the motion of a molecule is restricted to the fact that it finds an adjacent (target) site empty. In a square lattice, a molecule can chose randomly one of the 4 neighbouring sites. But in the case of MC the movement of a molecule *sp* also depends on the move probability P_{sp} provided by user.

The probability P_{sp} can be related to the diffusion coefficient value D_{sp} using the equation [10]

$$P_{sp} = 4D_{sp}\frac{\Delta t}{\Delta x^2} \qquad \text{(S1)}$$

Eq. (S1) helps one to decide if a particular molecule can move with the defined parameters Δx and Δt as was done in [1, 11] and in the off lattice methodology described in [10].

Considering the above, this section presents a comparison of 2D diffusion simulation of kMC (implemented in Fortran 90) and MC (the algorithm is described in [1,3,4], and it was implemented in MATLAB R2011a, The MathWorks, Natick, MA).

For this, the diffusion of tracer molecules is followed in a 100x100 square lattice in the presence of crowder' molecules, using Δx=1 (dimensionless). The (dimensionless) time step used in MC is Δt=1, with a move probability of P_{tracer}=1 for the tracer.

In the case of kMC, we use a diffusion coefficient equivalent to the above parameters Δx, Δt and P_{tracer} provided for the MC simulation (Eq. S1), i.e. $D_{sp} = 1/4$ (dimensionless).

Assuming periodic boundaries and that all molecules (tracers and crowders) occupy one single site, the diffusion simulation of tracer molecules (which occupy 0.5% of the total lattice space) is carried out at different crowding conditions (only one type of crowder is considered). All molecules are randomly allocated.

In order to reproduce the mean squared displacement (MSD) of tracer molecules reported in the Fig. 1A of [4], we assume that crowders are immobile. The relative error estimated between the average MSD computed (after 50 repetitions) by kMC and MC is calculated as

$$error_{MSD}(t) = \frac{norm\left(MSD_{kMC}(t) - MSD_{cLBM}(t)\right)}{MSD_{kMC}(t)}100 \qquad \text{(S2)}$$

Fig. S1A shows a very good agreement between MSD_{MC} and MSD_{kMC}, where the error estimated between them is less than 1% for all the crowding conditions tested (crowders occupying 0, 10, 20, 30, and 40% of the total space).

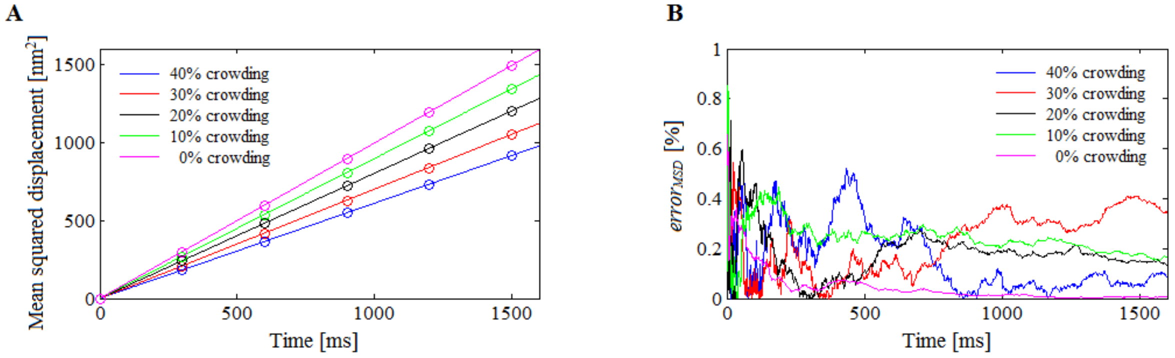

Figure S1 (A) Mean squared displacement of tracer molecules at different crowding concentration. (B) Relative error of the tracer's MSD estimated by MC−kMC. Tracer molecules represent 0.5% of the lattice area, immobile crowders represent: 0, 10, 20, 30, and 40% of the lattice. The colour lines indicates the MSD computed by kMC, while the circles represent the MSD computed by MC for each condition tested.

References

[1] Vilaseca E, Isvoran A, Madurga S, Pastor I, Garces JL, Mas F: **New insights into diffusion in 3D crowded media by Monte Carlo simulations: effect of size, mobility and spatial distribution of obstacles.** Phys Chem Chem Phys 2011, **13:**7396–7407.

[2] Vilaseca E, Pastor I, Isvoran A, Madurga S, Garces JL, Mas F: **Diffusion in macromolecular crowded media: Monte Carlo simulation of obstructed diffusion vs. FRAP experiments.** Theor Chem Acc 2011, **128:**795–805.

[3] Berry H: **Monte Carlo simulations of enzyme reactions in two dimensions: fractal kinetics and spatial segregation**. Biophys J 2002, **83:**1891–1901.

[4] Saxton MJ: **Lateral diffusion in an archipelago. The effect of mobile obstacles.** Biophys J 1987, **52:** 989–997.

[5] Grima R, Schnell S: **A systematic investigation of the rate laws valid in intracellular environments**. Biophys Chem 2006, **124:**1–10.

[6] Schnell S, Turner TE: **Reaction kinetics in intracellular environments with macromolecular crowding: simulations and rate laws.** Prog Biophys Mol Biol 2004, **85:**235–260.

[7] Reese JS, Raimondeau S, Vlachos DG: **Monte Carlo algorithms for complex surface reaction mechanisms: Efficiency and accuracy.** J Comput Phys 2001, **173:**302–321.

[8] Hari B, Goujon F, Theodoropoulos C: **Integrated multi-scale models for microreactor simulation and design.** AIDIC Conference Series 2009, **9:**167–176.

[9] Fragkopoulos IS, Theodoropoulos C: **Modelling of electrochemically promoted systems.** Electrochim Acta 2014, **150:**232–244.

[10] Ridgway D, Broderick G, Lopez-Campistrous A, Ru'aini M, Winter P, Hamilton M, Boulanger P, Kovalenko A, Ellison MJ: **Coarse-grained molecular simulation of diffusion and reaction kinetics in crowded virtual cytoplasm.** Biophys J 2008, **95:**3748–3759.

[11] Soula H, Care B, Beslon G, Berry H: **Anomalous versus Slowed-Down Brownian Diffusion in the Ligand-Binding Equilibrium.** Biophys J 2013, **105:**2064–2073.
